# Correction to: Genistein inhibits stemness of SKOV3 cells induced by macrophages co-cultured with ovarian cancer stem-like cells through IL-8/STAT3 axis

**DOI:** 10.1186/s13046-021-01958-y

**Published:** 2021-05-04

**Authors:** Yingxia Ning, Weifeng Feng, Xiaocheng Cao, Kaiqun Ren, Meifang Quan, A. Chen, Chang Xu, Yebei Qiu, Jianguo Cao, Xiang Li, Xin Luo

**Affiliations:** 1 Department of Gynaecology and Obstetrics, The First Affiliated Hospital of Guangzhou Medical University, Guangzhou, 510120 China; 2 The First Affiliated Hospital of Jinan University, Guangzhou, 510632 China; 3 Department of preclinical medicine, Medical College, Hunan Normal University, Changsha, 410013 China; 4 Department of Pharmaceutical Science, Medical College, Hunan Normal University, Changsha, 410013 China; 5 Key Laboratory of Study and Discover of Small Targeted Molecules of Hunan Province, Changsha, 410013 China

**Correction to: J Exp Clin Cancer Res 38, 19 (2019)**

**https://doi.org/10.1186/s13046-018-1010-1**

Following publication of the original article [1], the authors identified minor errors in image-typesetting in Fig. 9; specifically in the panels shown in Fig. 9a and d as follows:

- Figure 9a: a ruler has been added alongside the fragments of the tumor
- Figure 9d: corrected the panel displaying HE straining, GEN (50 μM) (−) and Ad-shSTAT3 (+) (*top row, middle-right*)

**Fig. 9:**
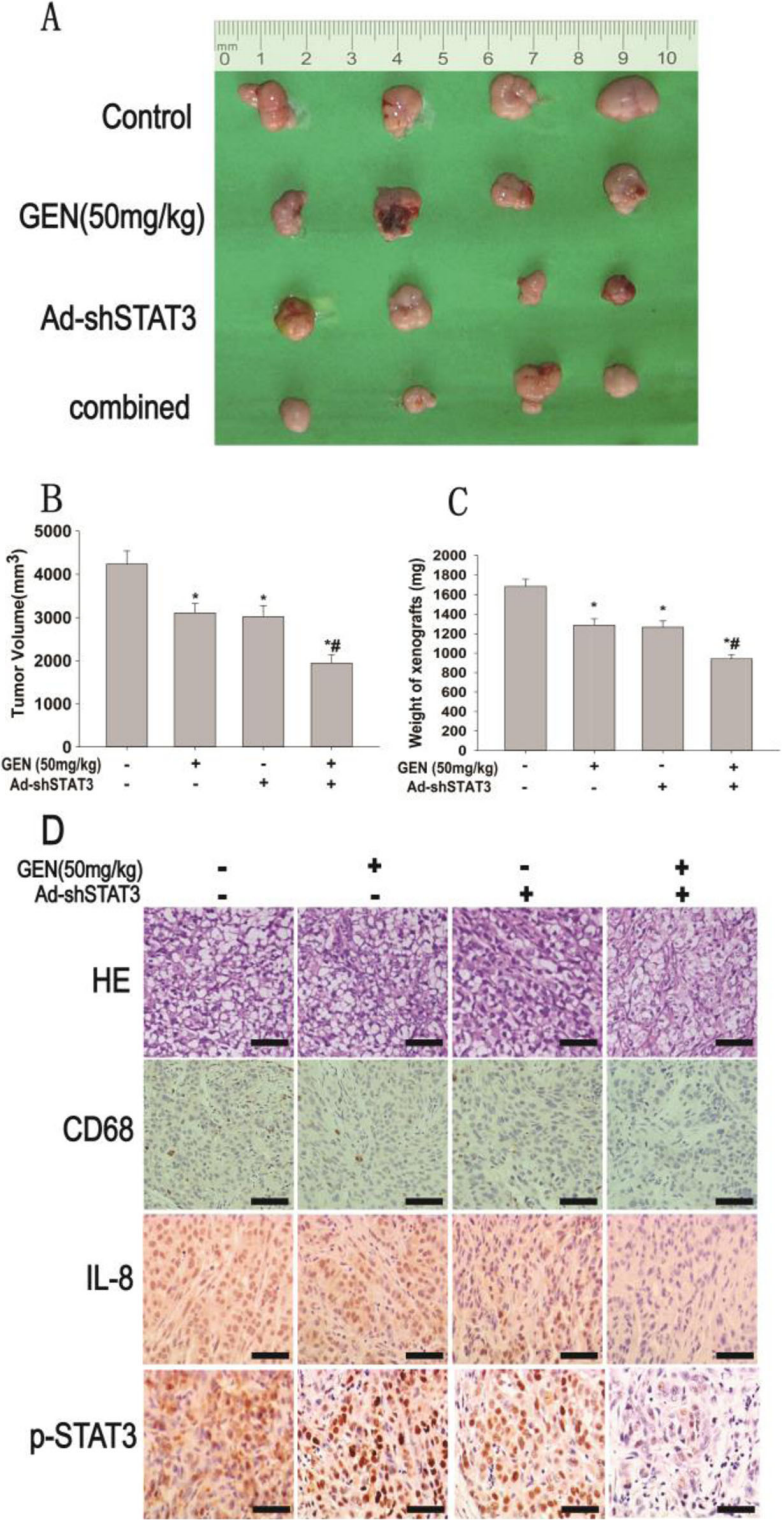
Combination of GEN and STAT3 shRNA inhibited xenograft growth by co-injection of SKOV3-derived OCSLCs and THP-1 macrophages. The nude mouse xenograft model using co-injection with OCSLCs/THP-1 macrophages was treated with GEN (50 mg/kg) and Ad-shSTAT3 alone or in combination. The size (**a**) volume (**b**), weight (**c**), histological examination (HE staining) and the expression of CD68, IL-8 and p-STAT3 (immunohistochemical staining) (**d**) of xenografts were shown (scale bar, 100 μm). *P < 0.05, vs the model control group; ^*#*^*P < 0.05*, vs treated with GEN (50 mg/kg) or Ad-shSTAT3 alone (means±SD, *n* = 4)

The correction does not have any effect on the results or conclusions of the paper. The original article has been corrected.
